# Gellan Gum in Wound Dressing Scaffolds

**DOI:** 10.3390/polym14194098

**Published:** 2022-09-30

**Authors:** Zizo Feketshane, Sibusiso Alven, Blessing Atim Aderibigbe

**Affiliations:** Department of Chemistry, University of Fort Hare, Alice Campus, Alice 5700, Eastern Cape, South Africa

**Keywords:** biopolymers, gellan gum, wound healing, hydrogels, films, nanofibers

## Abstract

Several factors, such as bacterial infections, underlying conditions, malnutrition, obesity, ageing, and smoking are the most common issues that cause a delayed process of wound healing. Developing wound dressings that promote an accelerated wound healing process and skin regeneration is crucial. The properties of wound dressings that make them suitable for the acceleration of the wound healing process include good antibacterial efficacy, excellent biocompatibility, and non-toxicity, the ability to provide a moist environment, stimulating cell migration and adhesion, and providing gaseous permeation. Biopolymers have demonstrated features appropriate for the development of effective wound dressing scaffolds. Gellan gum is one of the biopolymers that has attracted great attention in biomedical applications. The wound dressing materials fabricated from gellan gum possess outstanding properties when compared to traditional dressings, such as good biocompatibility, biodegradability, non-toxicity, renewability, and stable nature. This biopolymer has been broadly employed for the development of wound dressing scaffolds in different forms. This review discusses the physicochemical and biological properties of gellan gum-based scaffolds in the management of wounds.

## 1. Introduction

There are about 165 million cases of various types of wounds worldwide each year that require treatment. Surgical injuries occupy a vast majority of the wounds (103 million), followed by lacerations (20 million), diabetic ulcers (11 million), and burn wounds (10 million) [1]. The delayed process of wound healing is one of the major challenges in the treatment of wounds, and the wounds that suffer from delayed wound recovery mechanisms are called chronic wounds. The factors that contribute to a retarded wound healing process include underlying physiological conditions (i.e., diabetes mellitus and cancer), infections, obesity, smoking, and diet [2]. Wound dressings are materials that are broadly used for the management of various wounds. These materials are considered ideal when loaded with bioactive agents, exhibit good antibacterial activity, excellent biocompatibility, biodegradability, good bioadhesive properties, non-toxicity, provide a moist environment, promote cell migration and attachment, and provide gaseous permeation [3,4]. Wound dressings are used in various forms, such as hydrogels, nanofibers, films, and sponges. There are four sequential phases of the wound healing process that can be targeted by wound dressing materials: hemostasis, inflammatory, proliferation, and maturation (remodelling) [5].

Hemostasis is the first immediate response of the body to a wound. This phase prevents excessive bleeding through a blood clotting cascade. Platelet activation significantly improves the development of a fibrin clot that plugs the injured site [6]. The inflammation phase takes place concurrently or immediately after the hemostasis. This phase is vital for preventing microbial infection during the early stage of the wound healing process. Migration and influx of immune cells such as neutrophils, lymphocytes, growth factors (e.g., fibroblast growth factor, platelet-derived growth factor), and monocytes (which are differentiated into macrophages) occur in this phase. Some of these immune cells help in the removal of dead cells and debris, bacteria, and foreign substances [7,8]. In the proliferation phase, there is migration and proliferation of keratinocytes, endothelial cells, and fibroblasts at the wound bed. Granulation tissue development, angiogenesis, and re-epithelialization are the major events that take place in this phase [9,10]. The maturation phase starts 2–3 weeks after the injury and lasts for years (two or more). The main purpose of this phase is to form new epithelium and to promote tissue maturation by the apoptosis of extra endothelial cells, inflammatory cells, and fibroblasts [11,12].

The materials that are used for the treatment of wounds or targeting wound healing phases are widely produced from polymers. Polymers are categorized into two groups depending on their origin: natural and synthetic polymers. Natural polymers that are also known as biopolymers are extracted from microbial, plant, and animal sources. These polymers have attracted much attention from biomedical researchers due to their interesting properties that include non-toxicity, good cytocompatibility, closely simulating the original extracellular matrix (ECM), non-immunogenicity, and being readily available. Examples of natural polymers are cellulose, alginate, chitosan, collagen, gelatin, polymer gums (gellan gum, xanthan gum, dextran, tragacanth gum, karaya gum, guar gum, etc.) [13,14,15]. The limitations of natural polymers are poor mechanical performance and can be improved by combining them with synthetic polymers. The synthetic polymers that are frequently used in the preparation of hybrid-based wound dressings include poly (vinyl alcohol) (PVA), poly (vinyl pyrrolidone) (PVP), poly (hydroxyethyl methacrylate) (PHEMA), polyglycolic acid (PGA), polylactide (PLA), polycaprolactone (PCL), and poly (lactic-co-glycolic acid) (PLGA) (Table 1) [16]. It has been reported that natural polymers are mostly preferred for wound management over synthetic polymers due to their properties, such as biodegradability, non-toxicity, and biocompatibility. Moeini et al. reported that natural and synthetic polymers can both be used in the preparation of wound dressing. However, biodegradability and biocompatibility are interesting physicochemical features of natural polymers [17].

Nevertheless, the natural polymers’ drawback is their mechanical properties. The combination of natural and synthetic polymers produces improved mechanical properties. Okur et al. developed a film-based wound dressing using natural polymers (carbopol, chitosan, and sodium alginate,). Pure natural polymers demonstrated promising antibacterial activities against some bacterial strains. It has been reported that a good wound dressing should be able to stretch, but the wound dressings consisting of sodium alginate and chitosan are brittle without a plasticizer because both polymers exhibit high crosslink polymer properties. When PEG (polyethylene glycol) and PPG (propylene glycol) (synthetic polymers) were used as plasticizers, improved flexibility and stretchability (mechanical properties) of the wound dressings were reported. The films with PEG as their plasticizer were more ductile than the ones with PPG; this is due to PEG’s low molecular weight [18]. This review is focused on wound dressing scaffolds prepared from gellan gum with unique properties, such as ductility, thermoresponsive nature, non-toxicity, and biocompatibility. The unique features of gellan gum when used in wound dressings make it interesting to review. This review highlights the several advantages of gellan gum-based wound dressings.

**Table 1:** Natural and synthetic polymers’ advantages and disadvantages.

Polymers	Advantages	Disadvantages	References
**Natural** (Alginates, starch, cellulose, pectin, gums, chitin, etc.)	Nontoxic, biodegradable, biocompatible, economical, abundant in nature, and easily metabolized from the body.	Poor mechanical properties, adhesive strength, and extraction process expensive.	[19,20,21,22,23,24]
**Synthetic** (Polyethylene glycol, polyesters, poly lactic acid, polyurethanes, polystyrene, etc.)	Chemical, structural, flexibility mechanical properties are easily improved/controllable, excellent hydrophilic, and	Possibility of toxicity, slow degradation, and poor biocompatibility.	[25,26,27,28,29]

## 2. Classification of Wound Dressings

Wound dressing materials are commonly classified into five categories: traditional dressings, skin substitutes, dermal grafts, interactive dressings, and bioactive dressings (Table 2). Traditional wound dressings, also known as passive dressings, are used to keep the wound from contamination or foreign substances, absorb wound exudate, stop bleeding, and cushion the lesion [30,31]. Examples of traditional dressings are bandages, gauze, plaster, and wool dressings. However, these dressings suffer from limitations, including damage to the skin during removal and wound exudate leakage that can cause bacterial infections [16]. Skin substitutes are made up of tissue-engineered structures, typically produced from cell-seeded materials, and they are useful for skin tissue regeneration. Skin substitutes include TransCytes, Apligraf, and OrCel. The disadvantages of skin substitutes are that they are expensive, have limited shelf life, can cause the transmission of diseases to the host and wound infection, and can be rejected by the body [32,33]. Dermal grafts are the most important materials in the field of plastic surgery and dermatology. Examples of dermal grafts are allografts, autografts, and acellular xenografts. Dermal grafts are employed in traumatic wounds, defects after oncologic resection, burn reconstruction, contracture release, vitiligo, congenital skin deficiencies, and scar hair restoration. Nonetheless, they are not appropriate for the treatment of complex wounds (i.e., deep spaces and conditions with exposed bones) [34,35].

Interactive wound dressings provide a moist environment to accelerate the wound healing process, exhibiting good water vapour transmission, and enhancing granulation and re-epithelization. Examples of interactive dressings include gels, films, membranes, composites, and sprays [36]. Some of these dressings can be loaded with therapeutic agents to produce bioactive wound dressing materials. Bioactive wound dressings, such as sponges, hydrocolloids, foams, wafers, hydrogels, nanofibers, films, and collagens are biocompatible, biodegradable, and can act as drug delivery systems for bioactive agents, such as antibiotics, growth factors (GFs), nanoparticles, and vitamins with the enhanced process of wound healing [37]. Although hydrogels and nanofibers are other kinds of wound dressings, they can be encapsulated with therapeutic agents and exhibit the controlled drug delivery of bioactive agents, making them bioactive wound dressings [38].

**Table 2:** Classes of wound dressings.

Classification of Wound Dressings	Commercial Examples:	Advantages	Disadvantages	References
**Traditional**	Bandages, cotton wool, plaster, and gauze	Prevent bacteria contamination and absorb a lot of exudates.	Causes dehydration of the wound and tends to adhere to the wound surface resulting in pains upon removal.	[39,40,41]
**Biological**	Epidermal and dermal skin replacements, skin substitutes, and grafts	Regenerate the lost tissue.	Prone to infection transmission, the possibility of host rejection, and the formation of hypertrophic scars.	[42,43,44]
**Interactive**	None	Provide a moist environment for the wound.	They are semi-occlusive and have poor mechanical stability.	[45,46,47]
**Bioactive or artificial dressings**	Foams, wafers, hydrogels, and transdermal patches	Loaded with antimicrobial agents, biocompatible and biodegradable; They exhibit superior features when compared to other classes of wound dressings.	-	[48,49,50,51]

## 3. Properties of Gellan Gum

**Figure 1:**
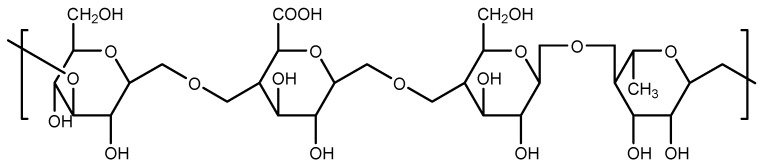
Molecular structure of gellan gum.

Several biomedical researchers have reported various forms of wound dressings that are based on polymer gums. The word ‘’Gum’’ is used to describe a group of naturally occurring polymers due to their capability to produce either viscous solution or gel. These polymers are widely studied because of their biodegradable, biocompatible, and sustainable features [52,53]. Among them is gellan gum which is commercially formulated by bacterial exopolysaccharide fermentation from *Pseudomonas elodea* or *Sphingomonas elodea*, and it can also be excreted by *Sphingomonas paucimobilis* but with a lesser yield. This polymer gum possesses negatively charged, linear exopolysaccharides consisting of four repeating carbohydrates in the main chain that includes two D-glucose carbohydrates, one D-glucuronic acid, and one L-rhamnose (Figure 1) [54,55]. Gellan molecules are found in the form of double helices at low temperatures and in the form of random coils at high temperatures. The average molecular weight of gellan gum is approximately 500 kDa [55].

There are several properties of gellan gum useful for the design of wound dressing. It exhibits good tolerance to acid and heat stress during preparation [56], and is ductile, thermoresponsive, biodegradable, non-toxic, and biocompatible [57,58]. It also has good mucoadhesive properties and is considered pseudoplastic at a high shear rate. It is not degraded by an acidic environment and is resistant to enzymatic action [59]. It offers good processibility and a broad spectrum of suitable rheological and mechanical properties. It can preserve a bioactive agent from the low pH by producing gels. The best features of gellan gum include its gelling property, high efficiency, textures, and malleability [59]. In wound dressing application, it has shown promising results, such as the proliferation of cell growth, and a few studies have reported its good biocompatibility against human skin fibroblast cells [60]. 

## 4. Gellan Gum-Based Wound Dressings

### 4.1. Hydrogels

**Figure 2:**
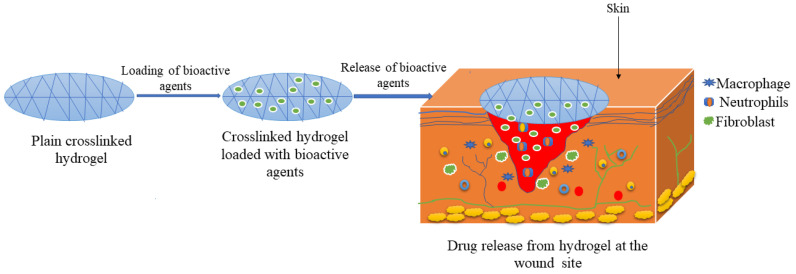
Schematic diagram of hydrogels.

Hydrogels are defined as scaffolds of hydrophilic polymers or lower molecular weight gelators that can retain a large amount of water and other biological fluids within their three-dimensional networks without dissolving [61]. These scaffolds have been widely used in wound healing applications due to their ability to load and retain bioactive agents within their network, offer a moist environment, and provide a desloughing and debriding capacity on necrotic and fibrotic tissue, biocompatibility, and good flexibility (Figure 2) [62]. Other advantages of hydrogels include their capability to soften the necrotic tissue on the wound bed, provide a soothing effect for overcoming pains, hydrate the wound surfaces and are non-adherent [63,64]. Although hydrogels demonstrate these interesting advantages, some of their shortcomings must be considered, such as poor mechanical stability in a swollen state, dehydration if they are not covered, and not being easy to secure, requiring a secondary dressing [65]. Nevertheless, there are many gellan gum-based hydrogels in pre-clinical studies that have shown promising outcomes in wound dressing applications.

Shukla et al. fabricated gellan gum–chitosan hybrid hydrogels enriched with ethanolic extract of *M. alba*, Apigenin, for the treatment of diabetic wounds [66]. The percent entrapment efficiency of gellan gum–chitosan hybrid hydrogels for Apigenin was 87.15 ± 1.20%, whereby the entrapment efficiency of the gellan gum and chitosan hydrogels was found to be 77.19 ± 0.5 and 79.08 ± 1.15%, respectively, suggesting that these hybrid hydrogels are good scaffolds for drug delivery. The swelling analysis showed that the gellan gum–chitosan hybrid hydrogels possess significantly higher water absorption properties when compared to the gellan gum and chitosan hydrogels, revealing that the gellan gum-based hybrid hydrogels are suitable for high exuding wounds. The in vitro drug release studies at physiological conditions (pH 7.4 and 37 °C) displayed an initial burst release of Apigenin from the gellan gum-based hybrid hydrogels followed by a sustained drug release for a prolonged period. The in vivo wound healing experiments using the Streptozotocin-induced diabetic wound model on Wister rats showed that the wound closure of Apigenin enriched-gellan gum-based hybrid hydrogel treated groups was significant with a faster wound healing process confirmed by a decreased epithelialization period when compared to the control group. The wounds were almost completely healed on day 18 for the Apigenin-loaded-gellan gum-based hybrid hydrogels while there was only 86.25% of wound healing on day 20 for the control group [66].

Shanmugapriya et al. formulated fucoidan-loaded gellan gum-alginate hybrid hydrogels [67]. The in vitro cytotoxicity studies using MTT assay displayed cell viability of more than 70% when the fucoidan-loaded gellan gum hybrid hydrogels were incubated with fibroblasts (L929 and NIH3T3 cells), suggesting that these hydrogel scaffolds are non-toxic and biocompatible. The in vitro antioxidant experiments utilizing DPPH scavenging assay revealed that fucoidan-loaded gellan gum hybrid hydrogels possess a high antioxidant scavenging activity when compared to the plain hybrid hydrogels, demonstrating their capability to overcome the prolonged inflammatory phase of the wound healing process. The in vivo wound healing studies showed a faster wound closure rate for the fucoidan-loaded gellan gum hybrid scaffolds group compared to other groups [67]. The gellan gum-chitosan hybrid hydrogels co-encapsulated with antibacterial drugs, tetracycline hydrochloride, and silver sulfadiazine, formulated by Zhang et al. showed excellent antibacterial activity against staphylococcus aureus (*S. aureus*) and Escherichia coli (*E. coli*) by displaying a high zone of inhibition in vitro [67]. These antibacterial results showed that gellan gum hybrid hydrogels loaded with antibacterial agents are suitable for treating clinical bacterial-infected wounds. 

Mohd et al. prepared gellan gum/montmorillonite (clay) hydrogels for skin regeneration and wound dressing applications [1]. The water vapour transmission rate (WVTR) values of these hydrogels ranged between 1106 and 1890 g m^−2^ d^−1^, which are appropriate for the fast wound healing process. The in vitro cytocompatibility studies revealed the non-cytotoxic effect of the hydrogels when incubated with human skin fibroblast cells (CRL2522) for 72 h. The gellan gum/montmorillonite hydrogels displayed a high zone of inhibition against *S. aureus* and *B. cereus* (Gram-positive bacteria), while there was no inhibition effect against *E. coli* and *K. pneumoniae* (Gram-negative bacteria) [1]. Pacelli et al. prepared gellan gum/clay (laponite) hydrogels loaded with an antibiotic (ofloxacin) for the treatment of wounds. The in vitro drug release experiments at physiological conditions showed that 1% *w*/*v* of laponite in gellan gum hydrogels exhibited a sustained drug release of ofloxacin for 8 h, suggesting that these hydrogels can significantly prevent infections in the wound bed. The cytotoxicity results of ofloxacin-loaded gellan/laponite hydrogels cultured with a human fibroblast cell line (WI-38) showed 81.5 ± 1.6% and 74.3 ± 1.3% cell viability after 24 and 96 h, respectively, indicating that these hydrogels are biocompatible [68]. 

Muktar et al. fabricated gellan gum-based hydrogels co-incorporated with virgin coconut oil and manuka honey for wound healing applications. The in vivo studies using the full-thickness wound model on Sprague-Dawley (SD) rats revealed significantly accelerated wound healing after 7 days of wound dressing by more than 50% with wound closure on the 7th and 11th day (67 ± 4% and 92 ± 4%, respectively). On day 14, the hydrogels co-incorporated with virgin coconut oil and manuka honey showed 98 ± 1% wound healing followed by the hydrogels incorporated with virgin coconut only and OPSITE (a commercial wound dressing) (95 ± 2% and 94 ± 2%, respectively) [69]. The pristine gellan gum/collagen hydrogels formulated by Ng et al. displayed early wound contraction in murine full-thickness burn wounds, reduced inflammation, and induced complete skin regeneration [70]. Xu et al. designed gellan gum-based hydrogels and cross-linked them with methacrylate to improve their mechanical strength. The mechanical compression moduli of the hydrogels ranged between 6.4 and 17.2 kPa, depending on the amount of methacrylate. The in vitro cell proliferation studies of the hydrogels showed the highest cell proliferation of NIH/3T3 fibroblasts and no dead cells were observed, suggesting that these scaffolds are non-cytotoxic and possess excellent biocompatibility [71]. 

Li et al. synthesized gellan gum/polyacrylamide hybrid hydrogels incorporated with magnesium ions. These hydrogels displayed increased tension strength from 86 to 392 kPa and elongation at break from 84 to 231% compared with plain gellan gum hydrogels, indicating good mechanical performance that is significant for wound dressing. The in vivo experiments using the rat full-thickness burn model showed that the gellan gum/polyacrylamide hybrid hydrogels incorporated with magnesium ions significantly accelerated wound closure in the case of the sustained release of magnesium ions in the wound beds [72]. Ozkahraman et al. designed gellan gum-based bilayer hydrogels loaded with an antibiotic, ampicillin. The scanning electron microscope (SEM) micrographs of the bilayer hydrogels showed porous structures with mean pore sizes that ranged between 268.6 ± 305 and 337.3 ± 179 µm. The in vitro antibacterial studies using the agar disc diffusion test showed that no inhibition zone was found in the ampicillin-free bilayer hydrogels while ampicillin-loaded hydrogels formed significant inhibition zones against *E. coli* and *S. aureus* cell-loaded agar plates, demonstrating that ampicillin-loaded hydrogels are promising wound dressing scaffolds for treating infected wounds [73].

Sebri and Amin formulated gellan gum-based hydrogels loaded with ibuprofen for wound dressing application. The mechanical experiments showed that the gellan gum hydrogel incorporated with 5.0% ibuprofen possessed interesting mechanical properties with the highest compressive strength of 200 ± 21 kPa and elastic modulus of 1820 ± 10 kPa. The WVTR value of the hydrogel incorporated with 5.0% ibuprofen was 867.5 ± 154 g m^−^^2^d^−^^1^, which is comparable with commercial wound dressing products. These hydrogels exhibited a slight antibacterial activity on *S. aureus* with an inhibition zone of 9.7 ± 1.15 mm, whereby cytotoxicity studies on human dermal fibroblast cells (CRL2522) confirmed that ibuprofen-loaded gellan gum hydrogels are biocompatible with the human cell line [74]. Muktar et al. prepared gellan gum hydrogels enriched with virgin coconut oil for wound treatment. The in vivo wound healing experiments using SD rats revealed a wound closure of 95 ± 2% after the 14th day when full-thickness wounds were treated with virgin coconut oil-enriched hydrogels while OPSITE wound dressing completed the wound closure of over 93 ± 4% on day 14. No skin irritation was observed during these experiments, indicating that the gellan gum is a safe biopolymer and a good material in wound healing applications [75].

Gellan gum hydrogels exhibit interesting properties that make them suitable candidates for the treatment of various wounds (e.g., burns and diabetic wounds). These hydrogel wound dressings exhibit moderate WVTR values that are appropriate to keep the wound bed moist for an accelerated wound healing process. The mechanical properties of gellan hydrogel dressings were improved by combining them with other polymers or incorporating them with cross-linking agents, making them excellent scaffolds that can be easily handled and applied during wound dressing. The in vitro cytotoxicity experiments of gellan gum hydrogels demonstrated high cell viability when incubated with various human skin cells, indicating non-toxicity and excellent cytocompatibility. Nevertheless, pure gellan gum hydrogels displayed no antibacterial properties toward Gram-positive and Gram-negative bacterial strains. This limitation was overcome by loading these hydrogels with various bioactive agents, such as antibiotics and essential oils. Interestingly, the in vivo wound healing studies showed fast wound closure of various wound models, such as burns and full-thickness wounds. These outcomes revealed that gellan gum hydrogels are promising ideal wound dressing materials for treating different types of wounds. 

### 4.2. Nanofibers

**Figure 3:**
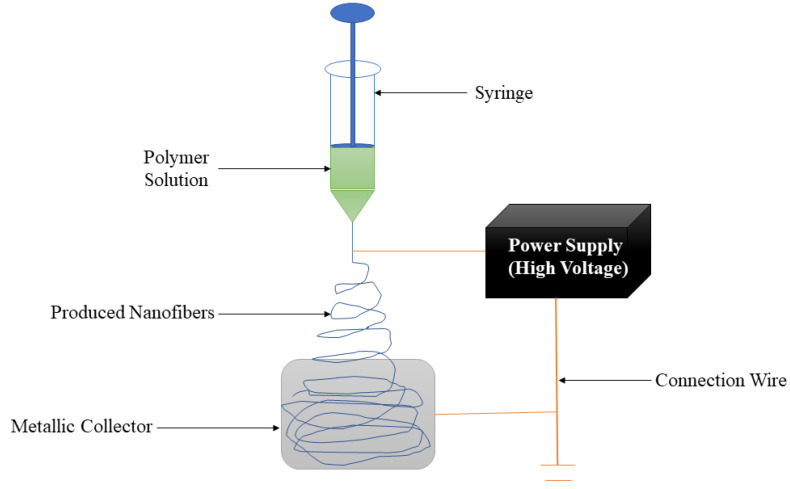
Fabrication of nanofibers via electrospinning technique.

Nanofibers are wound dressing materials that possess an average diameter of fewer than 1000 nanometers [76]. The nanofiber wound dressings are easily removed from the wound after their application. Electrospinning is a commonly employed technique for the fabrication of nanofibers. It is relatively flexible, simple, and produces nanofibrous scaffolds closely resembling the natural extracellular matrix (ECM) in size, scale, and structure [77,78]. The setup of the electrospinning technique is shown in Figure 3. Many prepared nanofibrous structures have been reported to be biocompatible with many skin cell types. Two important advantages of electrospun nanofibrous scaffolds are their large available surface area, efficient functionalization, and the possibility of formulating them from a broad variety of natural and synthetic polymers [79,80]. Other benefits of electrospun nanofibers that can contribute to skin regeneration and wound healing applications include high porosity, small diameter, gas permeation, and narrow diameter distribution [81,82]. Some researchers have reported the pre-clinical outcomes of gellan gum-based nanofibers for the treatment of wounds.

Mishra et al. fabricated gellan gum/PVA hybrid nanofibers incorporated with cinnamaldehyde (a phytoactive molecule present in cinnamon essential oil) using the electrospinning technique for the treatment of wounds [83]. The field emission scanning electron microscopy (FESEM) micrographs of cinnamaldehyde-loaded gellan gum-based hybrid nanofibers and pure gellan gum-based hybrid nanofibers displayed fibrous morphology with average diameters of 278.5 ± 57.8 nm and 204.03 ± 39.14 nm, respectively, indicating that these nanofibers mimic the extracellular matrix (ECM) that can support the cell growth. The in vitro drug release studies displayed a rapid release of cinnamaldehyde from nanofibers that can swiftly inhibit microbial growth. The in vitro antibiofilm experiments using XTT reduction assay showed that cinnamaldehyde-loaded gellan gum-based hybrid nanofibers effectively eradicated 50.45% and 89.29% of *Candida albicans* and *Candida glabrata*, respectively. The cinnamaldehyde-loaded gellan gum nanofibers exhibited superior antibacterial effects against *S. aureus* and *P. aeruginosa* when compared to the plain nanofibers, indicating their potential application as antibacterial wound dressing materials [83]. In addition, Vashisth and Pruthi designed electrospun gellan gum/PVA hybrid nanofibers for tissue engineering and wound healing applications. The in vitro cytocompatibility studies using MTT assay showed high cell proliferation or viability of human dermal fibroblast cells (3T3L1) when incubated with gellan gum/PVA hybrid nanofibers, suggesting good biocompatibility [84]. 

Vashisth et al. formulated amoxicillin-loaded gellan gum/PVA hybrid nanofibers via the electrospinning method for wound treatment. FESEM micrographs of electrospun amoxicillin-loaded gellan gum/PVA hybrid nanofibers displayed a smooth, bead-free, uniform nonwoven structure with a mean fibre diameter of 60 ± 37 nm. The in vivo wound closure assay of pure and amoxicillin-loaded gellan gum/PVA hybrid nanofibers on the dorsal excised full-thickness wounds of rats exhibited a significant wound contraction rate than the control (untreated wounds) [85]. The electrospun gellan gum/PVA hybrid nanofibers formulated by Vashisth et al. displayed uniform bead-free nanofibers with an average diameter of 40 ± 15.8 nm, mimicking biological ECM. Furthermore, the low applied voltage and longer tip-to-collector distance resulted in the origin of an almost similar type of nanofiber while the high flow rate did not display much influence on the gellan gum/PVA hybrid nanofibers [86,87].

Palumbo et al. formulated electrospun gellan gum/PVA hybrid nanofibers for treating wounds. Their pore size was 1.5 ± 0.6 µm with a homogeneous matrix composed of randomly deposited long nanofibers. The in vitro cytotoxicity revealed excellent biocompatibility and non-toxicity of nanofibrous scaffolds when incubated with fibroblasts (NIH 3T3 cells) for 7 days [88]. There are very few pre-clinical reports of gellan gum-based nanofibers that can be used for the treatment of wounds. It was necessary to incorporate gellan gum with PVA to generate spinnable polymer blends for all the reported electrospun gellan gum nanofibers. The FESEM or SEM micrographs of gellan gum-based nanofibrous scaffolds were bead-free, smooth with uniform structures, and average fibre diameter in the nanometers range, mimicking the ECM that can support the cell growth of various human skin cells. The in vitro cytocompatibility results using human skin cells showed that gellan gum-based nanofibers are biocompatible and non-toxic wound dressing materials. Most reports of gellan gum-based nanofibrous scaffolds developed have not been evaluated in vivo. The promising results of gellan gum-based nanofibrous scaffolds reveal the need for more research on these scaffolds to fully understand their potential in wound healing.

### 4.3. Films and Membranes

**Figure 4:**
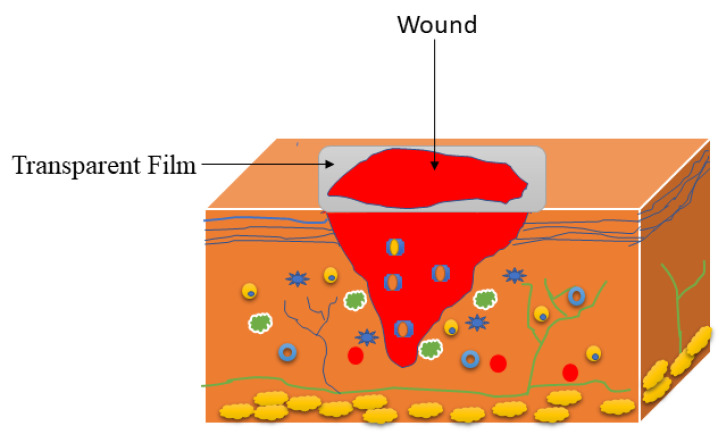
Film-based wound dressing.

Films are wound dressing scaffolds fabricated to permit the diffusion of carbon dioxide, oxygen, and water vapour from the injury, and are useful for autolytic removal of damaged tissues from the wounds [89,90]. These scaffolds exhibit several interesting properties that make them useful in the field of wound dressing, including high flexibility and elasticity leading to their ability to be altered to any shape of interest, and they do not need any additional tapping. Furthermore, the wound can be assessed during wound recovery by removing the films because of their transparency (Figure 4), thus avoiding discomfort in a patient [91]. Film wound dressings are appropriate for shallow, epithelizing, and superficial lesions that have low exudates. Films that are based on gellan gum have been reported as potential wound dressing scaffolds. Ismail et al. prepared gellan gum films incorporated with titanium dioxide nanoparticles using the evaporative casting method for wound dressing applications [92]. The antibacterial studies showed the inhibition zone of TiO_2_ nanoparticle-loaded biofilm was 9 ± 0.25 mm and 11 ± 0.06 mm, against *S. aureus* and *E. coli*, respectively, which was good antibacterial activity while the plain films did not show any antibacterial effects. The in vivo wound healing experiments on Sprague-Dawley (SD) rats revealed a complete wound closure of 92% for TiO_2_ nanoparticle-loaded biofilms whereas this was only 80% for gellan gum biofilm on the 14th day, suggesting that TiO_2_ nanoparticle-loaded biofilms possess the highest wound closure rate than the pristine gellan gum film [92]. Furthermore, the TiO_2_ nanotube-incorporated gellan gum films prepared by Razali et al. exhibited excellent biocompatibility against 3T3 mouse fibroblast cells and resulted in the accelerated wound healing process of the open excision type lesions in the SD rat model [93].

Ismail et al. reported gellan gum film loaded with TiO_2_ nanotubes for skin tissue regeneration. The in vitro cytotoxicity studies of gellan gum film wound dressings exhibited a high rate of cell proliferation with no sign of toxicity against 3T3 mouse fibroblast cells, revealing excellent biocompatibility of gellan gum films [94]. Norfloxacin-loaded gellan gum films fabricated by Ismail et al. showed superior antibacterial efficacy against *S. aureus* and *E. coli* bacterial strains depending on the rate of drug release of norfloxacin; higher concentration of the released antibiotics resulted in stronger antibacterial effects [95]. Azam et al. formulated gellan gum films enriched with Manuka honey for wound dressing applications. The water vapour transmission rate (WVTR) of gellan gum films loaded with 10% Manuka honey was 1145 ± 175 g m⁻^2^d⁻^1^ which is comparable with the commercially available wound dressing products. The WVTR is good for suitable gaseous permeation and moderate moisture for the acceleration of the wound healing process [96]. The WVTR values of gellan gum film wound dressings prepared by Ismail et al. using the film casting technique were 422 ± 113 and 987 ± 113 g m⁻^2^d⁻^1^ for GELZANTM gellan gum film and KELCOGEL^®^ gellan gum film, respectively, which were also suitable for the acceleration of wound healing, especially KELCOGEL^®^ gellan gum film [97].

Gellan gum films containing virgin coconut oil that were formulated by Ismail et al. displayed non-toxicity to human skin fibroblast cells (CRL2522) with limited cell growth after incubation for 3 days, resulting from the hydrophobic influence of the surface of gellan gum films. The in vitro antibacterial analysis exhibited superior antibacterial activity against *S. epidermidis* and *S. aureus* (Gram-positive) and *P. mirabilis* and *P. aeruginosa* (Gram-negative) for films loaded with virgin coconut oil compared to the free virgin coconut oil, indicating that these materials are useful for treating bacterial-infected wounds without inducing any significant cytotoxic effects [98]. Lee et al. fabricated gellan gum films for wound management. The mechanical characterization of gellan gum films displayed high tensile strength that ranged between 43.2 ± 11.1 and 52.4 ± 2.3, MPa, revealing that these films are compatible with the human skin and can be easily handled during wound dressing application. The in vivo studies demonstrated that in the first few days after treatment, the wound healing was superior when compared to the control (Duoderm) [99]. 

Gellan gum-based films exhibited interesting features of an ideal wound dressing, making them promising candidates for wound healing. Gellan gum films showed excellent mechanical properties, good cytocompatibility, non-toxicity, and moderate WVTR with the capability to accelerate the wound healing process. Nevertheless, the pristine films demonstrated no antibacterial activity, making them not suitable for treating bacterial-infected wounds. There are also modified methods that have been used to increase the bacteriostatic properties of gellan gum by chemical oxidization. However, this approach decreases the mechanical properties which may result from the reduction in molecular weight during the oxidation process [100]. The loading of antimicrobial agents is a good approach to enhancing the antibacterial effects of gellan gum films [101,102]. 

### 4.4. Other Gellan Wound Dressing Scaffolds

The gellan gum-based wound dressing scaffolds have also been formulated in other forms of wound dressings, such as sponges, bandages, foams, wafers, and transdermal patches depending on the type of wound. Song et al. prepared gellan gum/collagen sponges for tissue regeneration applications [103]. The SEM images of sponges displayed porous morphology with an average pore of 114 ± 29.24 μm, indicating their ability to promote cell migration and proliferation, high water absorption capacity, and permeation of gases. The in vitro antioxidant studies using the DPPH (1,1-diphenyl-2-picrylhydrazyl) free radical procedure showed that the gellan gum/collagen sponge was 1.25 times more active than plain gellan gum sponges which can be attributed to the presence of collagen, suggesting that these scaffolds can be potential therapeutics for the management of wounds in the inflammatory phase. Furthermore, the cytotoxicity studies showed high cell adhesion and proliferation of NIH/3T3 fibroblasts when incubated with gellan gum/collagen sponges, demonstrating excellent biocompatibility and non-toxicity which are the properties of an ideal wound dressing material [103]. The gellan gum-based 3D printed scaffolds developed by Yua et al. demonstrated good potential application in wound dressing due to their biodegradation behaviour that can be tailored to allow for controlled drug delivery [104]. Reczynska-Kolman formulated composite wound dressing based on gellan gum (GG) and a blend of gellan gum and alginate, containing lipid nanoparticles incorporated with antibacterial peptide-nisin. The in vitro antimicrobial analysis using agar diffusion tests showed significant bacteria growth inhibition zones against *S. pyogenes*, indicating the capability to be a potential antibacterial wound dressing. There are few research reports on other scaffolds of gellan gum for the management of wounds, thus there is substantial further research required on gellan gum wound dressing scaffolds [102]. 

## 5. Conclusions and Future Perspectives

Gellan gum-based wound dressing scaffolds demonstrate interesting properties that make them beneficial for wound healing. Those properties include moderate WVTR, good mechanical performance, excellent biocompatibility and non-toxicity, good biodegradability that can induce skin tissue regeneration, and controlled and sustained drug release profiles. However, pristine gellan gum scaffolds possess poor antibacterial and antioxidant activity, making them unsuitable for bacteria-infected wounds. These limitations have been overcome by loading bioactive agents (e.g., antibiotics, nanoparticles) and by chemical oxidation to improve the biological activities of gellan gum dressings. Due to the promising outcomes of gellan gum-based wound dressings in vitro and in vivo, more preclinical research studies are needed. There is currently no report on other forms of wound dressings prepared from gellan gum, such as bandages, patches, foams, etc., revealing that the design of gellan gum-based wound dressings is still in the infancy stage. There is no doubt that the design of more gellan gum-based wound dressing scaffolds will result in effective wound dressings that will reach clinical application for wound treatment soon, especially those loaded with therapeutic agents.
